# Impact of Age and Sex on Viral Load in Hepatitis C Virus Infection

**DOI:** 10.3390/v17010021

**Published:** 2024-12-27

**Authors:** Andrea Magri, Giulia Francesca Manfredi, Carlo Smirne, Silvia Pigni, Michela Emma Burlone, Mattia Bellan, Nicole Vercellino, Rosalba Minisini, Mario Pirisi

**Affiliations:** 1 Department of Translational Medicine, Università del Piemonte Orientale, 28100 Novara, Italy; andrea.magri@med.uniupo.it (A.M.); gf.manfredi01@gmail.com (G.F.M.); carlo.smirne@med.uniupo.it (C.S.); s.pigni98@gmail.com (S.P.); michela.burlone@med.uniupo.it (M.E.B.); mattia.bellan@med.uniupo.it (M.B.); nicole.vercellino@uniupo.it (N.V.); rosalba.minisini@med.uniupo.it (R.M.); 2 Nuffield Department of Medicine, University of Oxford, Oxford OX3 7JT, UK

**Keywords:** hepatitis C, HCV, age, sex, viral load, HCV genotype, sex hormones, liver fibrosis, estrogens, menopause

## Abstract

The determinants of hepatitis C virus (HCV) viral load remain incompletely understood and may differ in females, who are relatively protected from the consequences of HCV infection during their reproductive years. We aimed to evaluate how age affects the relationship between sex and viral load. *n* = 922 patients (males *n* = 497, median age 62 years), all naïve to direct antiviral agents, were studied. Females were older (median age 68 vs. 57, *p* < 0.001) and had a higher prevalence of genotype 2 (33% vs. 20%, *p* < 0.001) than males; there was no difference between sexes regarding the METAVIR stage. The median HCV RNA concentration was 1.017 × 10^6^ IU/mL (interquartile range, 0.286–2.400). Among males, the METAVIR stage was the strongest independent predictor of a high viral load (defined as the highest two quartiles), with advanced stages inversely associated with viral load (*p* = 0.008). In females, age was the only independent predictor, with women aged ≥55 years exhibiting higher loads (*p* = 0.009). These findings are consistent with data showing that estrogens exert an antiviral effect in in vitro models of HCV. Their declining levels after the menopause may explain the “catch-up” phase of HCV-related liver disease, observed in older women.

## 1. Introduction

Hepatitis C virus (HCV) infection affects an estimated 58 million people worldwide and remains a leading cause of chronic liver disease, cirrhosis, and hepatocellular carcinoma [1]. The clinical course of HCV infection varies widely among individuals and is influenced by a complex interplay of genetic, immune, and environmental factors. These factors impact individual susceptibility to HCV infection, the likelihood of spontaneous viral clearance, the progression to chronic liver disease, and the response to antiviral therapies [2].

With regard to genetic factors, polymorphisms in the interferon lambda 3 (IFNL3, previously known as IL28B) gene are strongly linked with spontaneous HCV clearance and responses to interferon-based therapies [3,4]. Additionally, specific human leukocyte antigen (HLA) alleles, such as *HLA-B27* and *HLA-DQB103*, have been linked to more robust immune responses against HCV, aiding in viral elimination in some instances [5,6]. Regarding the host immune response, natural killer (NK) cells, for example, are essential for early virus control [7], and variations in killer cell immunoglobulin-like receptors (KIR genes) can affect NK cell activity against HCV infection [8].

Lifestyle factors, including alcohol consumption, smoking, obesity, and metabolic syndrome, have been convincingly linked to worsening HCV-associated liver damage [9,10]. Co-infection with other viruses, particularly human immunodeficiency virus (HIV) [11] and hepatitis B virus (HBV) [12], accelerates liver disease progression in HCV-infected individuals and complicates treatment outcomes [13]. Age at the time of infection is an important determinant, as older individuals are more likely to develop chronic infection and advanced liver disease [14]. Gender also plays a role, with males generally experiencing more rapid progression to fibrosis and cirrhosis, potentially due to differences in immune response and hormonal influences [15].

Several viral factors also influence the risk of chronic infection and the efficacy of treatments. Among them, elevated baseline viral loads have been associated with decreased rates of spontaneous viral clearance and poorer response to interferon-based treatments. Although the advent of direct antiviral agents (DAAs) has largely mitigated the impact of high viral loads on treatment outcomes, the determinants of viral load in individual patients remain incompletely understood, as they were in the early days after the discovery of HCV [9,11,12,16,17,18].

In the present study, we seized the opportunity to analyze the variability of the HCV viral load in relation to demographic, anthropometric, and clinical parameters of patients proposed for antiviral treatment with DAAs, aiming to identify its predictors; specifically, we attempted to evaluate how age affects the relationship between sex and viral load.

## 2. Materials and Methods

### 2.1. Study Design

We conducted a single-center, observational, retrospective, cross-sectional study involving patients diagnosed with chronic hepatitis C referred to the liver clinic of an academic hospital in Northern Italy. Data collection covered patient assessments from January 2015 to January 2019.

### 2.2. Study Population

The initial study population consisted of a consecutive series of patients aged 18 years or older, referred to our center for the treatment of chronic hepatitis C. For each patient, we collected the following information, grouped into the following three categories:

- Demographic data: ethnicity, age, and sex;
- Anthropometric data: weight and height;
- Clinical data: HCV genotype, disease stage based on METAVIR score [19], HIV co-infection status, HCV RNA level, and serum creatinine levels. Patients with HIV co-infection included in this study were undergoing antiretroviral therapy.

Patients were excluded from this study if they presented with HBV co-infection (*n* = 14), HBV and hepatitis D virus (HDV) co-infection (*n* = 1), or decompensated cirrhosis (*n* = 3), defined as a history or presence of ascites, hepatic encephalopathy, esophageal variceal bleeding, and jaundice. Moreover, patients were excluded from the analysis if disease staging data (*n* = 26), serum creatinine levels (*n* = 4), or body mass index (BMI) values (*n* = 39) were missing.

This study was conducted in full accordance with Helsinki criteria and was approved by the local ethical committee, Comitato Etico Interaziendale Novara, https://comitatoetico.maggioreosp.novara.it/, IRB code CE081/2022 (accessed on 3 December 2024).

### 2.3. Statistical Analysis

The data were analyzed using Stata 18 statistical software (StataCorp, College Station, TX, USA). Categorical variables were reported as absolute values and percentages. For continuous variables, medians and interquartile ranges (IQRs) were used as measures of central tendency and dispersion. Categorical variables were compared across subgroups using the χ^2^ test, Pearson’s chi-squared test, or Fisher’s exact test, as appropriate. Continuous variables were compared between groups using the Kruskal–Wallis or the Mann–Whitney test, as appropriate. Logistic regression models were constructed to assess factors independently associated with high viral load. Statistical significance was set at *p* < 0.05 for all analyses.

## 3. Results

### 3.1. Characteristics of the Study Population

A total of 1009 patients were initially evaluated for this study, with 922 patients ultimately selected based on the inclusion and exclusion criteria outlined in the Section 2. As shown in Table 1, of the included patients, 497 (54%) were male, and 425 (46%) were female, with 857 patients (93%) being native Italians. The median age at treatment was 62.1 years (IQR 51.9–74.0), and a statistically significant difference was observed between sexes (*p* < 0.001): female patients had a higher median age of 68.4 years (IQR 57.9–75.9) compared to male patients, who had a median age of 56.6 years (IQR 49.5–68.4). An additional significant age distribution difference was evident when a cut-off age of 55 years was considered (*p* < 0.001): 56% of male patients were aged ≥55 years at treatment, while 44% were <55 years; in contrast, 80% of female subjects were aged ≥55 years, and only 20% were <55 years. BMI was significantly higher in male patients, with a median of 25.5 kg/m^2^ (IQR 23.2–28.3) compared to 24.4 kg/m^2^ (IQR 21.6–28.3) in female patients (*p* = 0.003). Serum creatinine levels also differed significantly by sex (*p* < 0.001), with a higher median of 0.81 mg/dL (IQR 0.71–0.93) in males compared to 0.67 mg/dL (IQR 0.59–0.78) in females.

**Figure 1:**
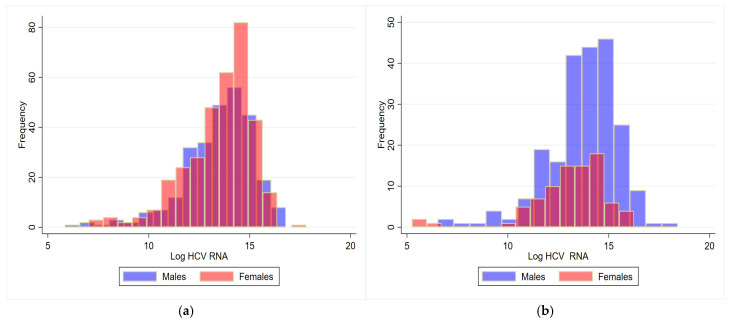
Distribution of log-transformed HCV RNA values of male and female patients, equal to or above 55 years (**a**) and below 55 years (**b**).

Figure 1 presents the distribution of log-transformed HCV RNA values of male and female patients, equal to or above 55 years (a) and below 55 years (b). The comparison between males and females belonging to the latter group demonstrated significantly higher geometric means of circulating HCV RNA in males (916,915 vs. 481,004 IU/mL, in males and females, respectively; *p* = 0.005), while there was no significant difference between sexes in the former group about this parameter (687,523 vs. 734,834 IU/mL; *p* = 0.616).

The distribution of HCV genotypes was also not uniform across sexes (*p* < 0.001). Genotype 1 was the most common for both sexes, found in 55% of females and 51% of males. Genotype 2 was more prevalent among females (33%) than males (20%), while genotypes 3 and 4 were more frequent in males, present in 18% and 10%, respectively, versus 8% and 3% in females. Additionally, HIV co-infection was significantly more common in males (9%) than in females (3%) (*p* < 0.001).

### 3.2. Univariate Analysis of Factors Associated with Viral Load

A univariate analysis was conducted to assess associations between various parameters (both clinical and demographic) and viral load levels. Patients were categorized into high viral load (*n* = 461) and low viral load (*n* = 461) groups, based on the median viral load value of 1,017,000 IU/mL. Among patients with low viral load, the median viremia was 286,300 IU/mL (IQR 109,200–570,100), while the high viral load group had a median value of 2,400,000 IU/mL (IQR 1,638,000–4,065,000). Patients of Italian origin were more frequently in the high viral load group (95%) compared to those in the low viral load group (91%) (*p* < 0.001). HCV genotype distribution also differed significantly between the two groups (*p* = 0.007): genotype 1 was more common in the low viral load group (57%) than in the high viral load group (49%), while genotype 2 was more prevalent in the high viral load group (31%) compared to the low viral load group (21%). Genotype 3 was similarly distributed between the two groups (14% in low viral load and 12% in high viral load), as was genotype 4 (7% in both groups) (Table 2).

Separate analyses for male and female patients revealed that, among males, only Italian origin was significantly associated with high viral load (*p* = 0.018). Among females, age ≥55 at the time of treatment was the only factor significantly associated with high viral load (*p* = 0.01) (Table 3).

### 3.3. Multivariate Analysis of Factors Associated with Viral Load

Three logistic regression models were developed to predict high versus low viral load (Table 4). In the total population model, disease stage and Italian origin were associated with viral load levels: specifically, Italian origin was linked to higher viral load (odds ratio (OR) of 1.61 and 95% confidence interval (CI) of 1.01–2.56, *p* = 0.043), while more advanced disease stages were associated with lower viral load (OR 0.90, 95% CI 0.81–0.97, *p* = 0.009). In the male-only model, disease stage remained a significant predictor of lower viral load (OR 0.85, 95% CI 0.75–0.96, *p* = 0.008), and Italian origin was again associated with higher viral load (OR 2.15, 95% CI 1.08–4.27, *p* = 0.029). In the female-only model, age ≥ 55 was the sole independent predictor, with older age associated with higher viral load (OR 2.05, 95% CI 1.20–3.51, *p* = 0.009).

## 4. Discussion

Several genetic, immunological, behavioral, metabolic, and demographic factors are associated with viral load, although many remain under investigation. In turn, viremia is a crucial factor influencing the clinical course of HCV infection [17]. Among the aforementioned demographic factors, sex is a significant modulator in the natural history of chronic hepatitis C [20]. In general, sex hormones can have protective effects against certain infections. Specifically, in HCV disease, being female is associated with higher rates of spontaneous clearance following acute infection and slower disease progression, compared to males [21].

In this study, we aimed to evaluate how age affects the relationship between sex and viral load. Our primary finding is that factors associated with viral load appeared to differ between males and females. In males, disease stage was the strongest independent predictor, with advanced stages associated with a lower likelihood of high viral load. Additionally, males born in Italy more frequently exhibited higher viral loads. In females, age was the sole independent predictor, with younger women more often presenting with lower viremias. Our data suggest that age may modulate the influence of female sex on viral load, potentially still due to the effects of sex hormones. These findings merit further examination in light of the current literature.

As described in the Section 2, this study included almost 1000 patients with a slight predominance of males. While the sample was sex-balanced, it was not age-balanced. Significant sex differences in age distribution were observed. Specifically, 56% of male patients were 55 years or older, while 44% were under 55. In contrast, 80% of female patients were 55 years or older, with only 20% under this threshold. Precise data on hormonal status, such as blood test results, were unavailable, so we used the 55-year cutoff as a proxy, assuming that women over this age are generally postmenopausal. This heterogeneity may reflect the increased likelihood of postmenopausal women seeking medical care due to hormonal changes, leading to higher diagnosis rates of chronic HCV at this stage.

Our sample consisted of 93% Italian natives, aligning well with the national population, as recent data from the Italian National Statistics Institute (updated to January 2024) showed that foreign nationals account for approximately 9% of Italy’s total population [22]. To reduce potential confounders, we excluded patients with HBV or HBV/HDV co-infections and those with decompensated cirrhosis. Indeed, HBV co-infection interferes with HCV viral replication, reducing HCV viral load [23,24], while viral replication significantly decreases in end-stage decompensated cirrhosis compared to compensated cirrhosis [25]. HIV-co-infected patients were not excluded, as they were all receiving antiretroviral therapy.

As previously reported, female sex offers a degree of protection against HCV infection: women are more likely to achieve spontaneous viral clearance, exhibit slower disease progression, and generally present with lower viremia levels than men [20]. However, this protective effect needs to be contextualized across different hormonal phases. Premenopausal women generally demonstrate reduced liver inflammation and better disease control, whereas postmenopausal women exhibit increased histological activity and a progression risk similar to age-matched men [26]. These observations suggest that sex hormones, particularly estrogens [27], are likely responsible for these beneficial effects, primarily through modulation of innate and adaptive immune cells [28,29].

Our study confirms that age at treatment is the only independent predictor of HCV viremia in females, with younger women more frequently presenting with lower viral loads. This finding supports an estrogen-mediated effect on viral replication inhibition, consistent with previous in vitro studies [30]. Additionally, estrogens have anti-fibrogenic effects and modulate metabolic parameters and oxidative stress, all of which influence liver disease progression [31]. Indeed, during reproductive age, women appear better able to control viral replication, resulting in lower liver necroinflammation and slower fibrosis progression than men [15].

Our results align with the notion that women of reproductive age may have relative protection against severe liver damage, followed by a “catch-up” phase after menopause, potentially due to declining estrogen levels. This assumes that viral load significantly influences the natural history of HCV. However, the relationship between viral load and disease progression remains debated, with findings varying across studies, probably due to differences in study design [32,33,34]. Whether higher fibrosis stages are associated with lower serum HCV RNA levels is also somewhat controversial [35,36]. Part of these discrepancies may be due to the impracticality of measuring viral load in parenchyma cells, likely a more appropriate index for evaluating HCV replication in hepatocytes, and possibly reduced in advanced stages of chronic hepatitis C. With specific regard to the findings we present here, other authors have failed to identify a clear association between viral load and demographic factors such as age and sex [37,38,39], possibly due to the underrepresentation of females or older individuals. Indeed, in many studies patients under 55 years were over-represented, limiting the ability to capture age-related variations in viremia.

However, our study also has several limitations. As a single-center study, the sample may be less diverse and less representative than a multicenter investigation. Its retrospective nature may also limit the applicability to current epidemiology. Furthermore, the cross-sectional design prevented us from tracking viral load trends over time. Finally, in the absence of specific data on hormonal status, we used age as a proxy, assuming women over 55 years were postmenopausal.

In conclusion, we demonstrated that viral load in chronic HCV infection is influenced by both sex and age. This finding represents a paradigmatic case of gender medicine and may hold value during discussions with young female patients, encouraging them to seek early treatment for hepatitis C.

## Figures and Tables

**Table 1:** Demographic and clinical characteristics of the study population. Categorical variables are described as absolute frequencies (*n*) with relative proportion (%), and continuous variables as medians with interquartile ranges (IQRs). Bold values denote statistical significance at the *p* < 0.05 level.

	Total (*n* = 922)	Male (*n* = 497)	Female (*n* = 425)	*p*
Age at treatment, years	62.1 [51.9–74.0]	56.6 [49.5–68.4]	68.4 [57.9–75.9]	**<0.001**
≥55 years, *n* (%)	618 (67)	277 (56)	341 (80)	
<55 years, *n* (%)	304 (33)	220 (44)	84 (20)	**<0.001**
Native Italians, *n* (%)	857 (93)	464 (93)	393 (92)	0.608
Body mass index, kg/m^2^	25.1 [22.4–28.3]	25.5 [23.2–28.3]	24.4 [21.6–28.3]	**0.003**
Serum creatinine, mg/dL	0.74 [0.64–0.87]	0.81 [0.71–0.93]	0.67 [0.59–0.78]	**<0.001**
Disease-stage METAVIR				
F0	157 (17)	75 (15)	82 (19)	
F1	168 (18)	93 (18)	75 (18)	
F2	143 (16)	77 (15)	66 (16)	
F3	147 (16)	80 (16)	67 (16)	
F4	307 (33)	172 (35)	135 (32)	0.543
HCV genotype				
1	487 (53)	254 (51)	233 (55)	
2	243 (26)	101 (20)	142 (33)	
3	120 (13)	87 (18)	33 (8)	
4	66 (7)	52 (10)	14 (3)	
Other, undetermined	6 (<1)	3 (<1)	3 (<1)	**<0.001**
HIV co-infection	63 (7)	45 (9)	14 (3)	**<0.001**
HCV RNA, ×10^3^ IU/mL	1017 (286–2400)	1033 (304–2554)	950 (268–2300)	0.226

Abbreviations: hepatitis C virus (HCV); human immunodeficiency virus (HIV); meta-analysis of histological data in viral hepatitis (METAVIR).

**Table 2:** Univariate analysis of factors associated with viral load (low/high). Categorical variables are described as absolute frequencies (*n*) with relative proportion (%), and continuous variables as medians with interquartile ranges (IQRs). Bold values denote statistical significance at the *p* < 0.05 level.

	Low Viral Load (*n* = 461)	High Viral Load (*n* = 461)	*p*
Age at treatment, years	62.0 [51.8–74.0]	62.3 [52.0–73.9]	0.86
≥55 years, *n* (%)	307 (67)	311 (67)	
<55 years, *n* (%)	154 (33)	150 (33)	0.83
Native Italians, *n* (%)	418 (91)	439 (95)	**0.01**
Body mass index, kg/m^2^	24.9 [22.0–27.9]	25.3 [22.7–28.4]	0.09
Serum creatinine, mg/dL	0.74 [0.63–0.87]	0.75 [0.64–0.87]	0.34
Disease-stage METAVIR			
F0	76 (16)	81 (18)	
F1	78 (17)	90 (20)	
F2	65 (14)	78 (17)	
F3	72 (16)	75 (16)	
F4	170 (37)	137 (30)	0.21
HCV genotype			
1	262 (57)	225 (49)	
2	99 (21)	144 (31)	
3	63 (14)	57 (12)	
4	32 (7)	34 (7)	
Other, undetermined	5 (1)	1 (<1)	**0.007**
HIV co-infection	24 (5)	35 (8)	0.18

Abbreviations: hepatitis C virus (HCV); human immunodeficiency virus (HIV); meta-analysis of histological data in viral hepatitis (METAVIR).

**Table 3:** Univariate analysis of factors associated with viral load (low/high) in females. Categorical variables are described as absolute frequencies (*n*) with relative proportion (%), and continuous variables as medians with interquartile ranges (IQRs). Bold values denote statistical significance at the *p* < 0.05 level.

	Low Viral Load (*n* = 218)	High Viral Load (*n* = 207)	*p*
Age at treatment, years	68.2 [55.4–76.0]	68.5 [59.9–75.8]	0.42
≥55 years, *n* (%)	164 (75)	177 (86)	
<55 years, *n* (%)	54 (25)	30 (14)	**0.01**
Native Italians, *n* (%)	198 (91)	195 (94)	0.20
Body mass index, kg/m^2^	24.1 [21.4–27.9]	24.7 [21.7–28.9]	0.33
Serum creatinine, mg/dL	0.67 [0.58–0.78]	0.67 [0.59–0.79]	0.70
Disease-stage METAVIR			
F0	45 (21)	37 (18)	
F1	38 (17)	37 (18)	
F2	27 (12)	39 (19)	
F3	35 (16)	32 (15)	
F4	73 (33)	62 (30)	0.44
HCV genotype			
1	128 (59)	105 (51)	
2	60 (28)	82 (40)	
3	20 (9)	13 (6)	
4	8 (4)	6 (3)	
Other, undetermined	2 (<1)	1 (<1)	0.11
HIV co-infection	6 (3)	8 (4)	0.59

Abbreviations: hepatitis C virus (HCV); human immunodeficiency virus (HIV); meta-analysis of histological data in viral hepatitis (METAVIR).

**Table 4:** Multivariate analysis to predict factors associated with viral load (high/low) in all (left), males (center), or females (right). Bold values denote statistical significance at the *p* < 0.05 level.

Factor	Total (*n* = 922)		Male (*n* = 497)		Female (*n* = 425)	
	OR (95% CI)	*p*	OR (95% CI)	*p*	OR (95% CI)	*p*
Biological sex	0.82 (0.64–1.05)	0.115				
Age at treatment	1.08 (0.80–1.46)	0.599	0.75 (0.52–1.08)	0.129	2.05 (1.20–3.51)	**0.009**
Native Italian	1.61 (1.01–2.56)	**0.043**	2.15 (1.08–4.27)	**0.029**	0.90 (0.47–1.74)	0.750
Body mass index	1.01 (0.99–1.04)	0.177	1.01 (1.10–4.27)	0.455	1.00 (0.99–1.03)	0.896
Serum creatinine	0.83 (0.67–1.03)	0.091	0.76 (0.41–1.41)	0.382	0.83 (0.66–1.04)	0.110
Disease-stage METAVIR	0.90 (0.81–0.97)	**0.009**	0.85 (0.75–0.96)	**0.008**	0.95 (0.88–1.02)	0.122
HCV genotype	0.98 (0.87–1.10)	0.729	0.98 (0.85–1.14)	0.824	0.95 (0.88–1.15)	0.616
HIV co-infection	1.30 (0.75–2.26)	0.351	1.17 (0.61–2.23)	0.631	1.92 (0.61–6.10)	0.262

Abbreviations: hepatitis C virus (HCV); human immunodeficiency virus (HIV); meta-analysis of histological data in viral hepatitis (METAVIR).

## Data Availability

Authors will make data and materials supporting the results or analyses presented in this paper available upon reasonable request.
